# Experiences of Older Adults With Mobile Phone Text Messaging as Reminders of Home Exercises After Specialized Manual Therapy for Recurrent Low Back Pain: A Qualitative Study

**DOI:** 10.2196/mhealth.7184

**Published:** 2017-03-30

**Authors:** Stina Charlotta Lilje, Ewy Olander, Johan Berglund, Eva Skillgate, Peter Anderberg

**Affiliations:** ^1^ Institute of Environmental Medicine Karolinska Institutet Stockholm Sweden; ^2^ Department of Health Blekinge Institute of Technology Karlskrona Sweden; ^3^ Scandinavian College of Naprapathic Manual Medicine Stockholm Sweden

**Keywords:** text messages, older adults, recurrent low back pain, manual therapy

## Abstract

**Background:**

Clinical experience of manual therapy for musculoskeletal pain is that patients often suffer from recurrent pain and disorders, but that they do not continue to perform their physical home exercises when they are free from symptoms. The chance of positive long-term effects of manual therapy would probably increase if patients were reminded that they are to continue to perform their exercises. Mobile phone text messaging (short messaging service, SMS) is increasingly used as an innovative intervention to remind patient to exercise. However, there are only a few studies on such interventions in the field of low back pain (LBP). Qualitative studies of patients’ experiences of receiving text messages as reminders of home exercises after manual treatment for recurrent LBP have to the best of our knowledge never been published.

**Objectives:**

The aim of this study was to explore older persons’ common experiences of receiving reminders of home exercises through mobile phone text messaging after specialized manual therapy for recurrent LBP.

**Methods:**

A total of 7 men and 8 women (67-86 years), who had sought specialized manual therapy (Naprapathic manual therapy) for recurrent LBP were included in the study. Individual one-way text messages as reminders of home exercises (to be performed on a daily basis) were sent to each patient every third day for 3 weeks, then once a week for another 2 weeks. Semistructured interviews with 2 broad, open-ended questions were held and data were analyzed with systematic text condensation, based on Giorgi’s principles of psychological phenomenological analysis.

**Results:**

The participants appreciated the messages, which were perceived as timely and usable, and also stimulated memorizing. The messages made the participants reflect on the aim of the exercise, value of being reminded, and on their improvement in pain. During the interviews, the participants created their own routines for continued adherence to the exercises.

**Conclusions:**

It seems plausible that mobile phone text messaging may serve as a useful tool for patient empowerment with regard to recurrent LBP in older persons. Further studies are needed to explore whether future compliance with the exercises will be as large if the participants are not being interviewed.

## Introduction

Low back pain (LBP) that interferes with normal life is common in the general population [1]. Positive effects from Naprapathic manual therapy (NMT) for LBP and other kinds of musculoskeletal disorders have been found in clinical trials. They include decreased pain, increased physical function, and perceived recovery, both in the short and in the long term [2-4]. The NMT concept is pragmatic; the patients’ knowledge about their disorders and their commitment to home exercises are believed to play an important role [5]. The aim of the home exercises is to improve the patients’ pain condition and to prevent recurrence, but clinical experience of patients with recurrent pain is that they do not continue to perform the exercises when their pain is not present anymore. If it is possible to increase patients’ adherence to home exercises, it may positively impact the long-term effects of the treatment, as well as increase the independence for patients and reduce costs. Therefore, it is necessary to give patients some empowerment over their improvement and to gain knowledge of how they experience being reminded of their home exercises.

Communication technologies are expanding and there are many areas in health care where it may be used for different purposes, for example, reminders of medication, fixing appointments in clinics, and pain assessment [6,7]. Mobile phone apps belong to a growing field of technological inventions that have had a positive impact on the outcomes of different interventions, their feasibility, and usability [8-10]. Responses to surveys can be more easily be given in real time using a mobile phone compared with postal surveys; the compliance is good, and it is also an inexpensive, time-efficient method.

Evaluations of the effects of mobile phone reminders for disease prevention, facilitation of self- management of long-term illnesses, and clinical and healthy behavior interventions are common, and the outcomes are positive in terms of significant improvement and differences regarding medication adherence, clinical management, and health-related behavior modification [11,14]. Mobile phones may also bridge gaps in health disparities; text messages were appreciated by the majority of participants in several studies and effects from text messaging exist across age, minority status, and nationality [11-13]. The majority of studies in this field are conducted in special health care settings; the most frequently studied patient groups are smokers, diabetics and patients with mental health disorders [15]. Research on mobile phone interventions for people with chronic pain in general and for LBP in particular is limited [16,17]; very little is known about preventive strategies and patient empowerment through mHealth, in LBP. Also, studies on vulnerable populations such as the elderly are requested [15]. In striving for increased health for patients with musculoskeletal pain, it is of interest to explore if using text messaging aimed to promote adherence to home exercises might be an appreciated delivery approach. Qualitative studies of patients’ common experiences of receiving text messaging about home exercises have never been performed before to the best of our knowledge.  The aim of this study was to explore the experiences of the elderly of a one-way reminder program using mobile phone text messaging aimed to promote adherence to home exercises following manual therapy for recurrent LBP.

## Methods

### Materials

The participants were 8 women (ages: 67, 73, 73, 77, 79, 82, 86, and 86) and 7 men (ages: 67, 71, 73, 75, 78, 81, and 82) who were consecutively treated for recurrent LBP in a clinic for specialized manual therapy (Table 1). Two patients (2 women aged 75 and 78 years) who were asked for participation did not want to be enrolled in the study. One of them did not possess a mobile phone, the other one was not interested in any participation. Another patient (a woman; 80 years) suffered from dementia.

**Table 1:** Demographics of the study participants or sample information.

Sample characteristics (n=15)		Values
**Gender**		
	Female	8
	Male	7
**Age**		
	Mean	76,7
	Range	67-86
**Living alone**		
	Yes	3
	No	12
**Previous profession**		
	Light physical load (illustrator, architect, office clerk, laboratory technician)	6
	Medium physical load (nurse, shop assistant, dentist, police, musician, house wife)	8
	Heavy physical load (farmer)	1
**Regularly exercising (walking with poles, golfing, going to the gym, doing gymnastics, felling)**		
	Yes	11
	No	4
**Number of treatments**		
	Mean	2,9
	Range	1-5

### Intervention

The profession NMT has been a part of the Swedish health and medical care system since 1994. It is defined as a system for specific examination, diagnosis, and manual treatment of soft and connective tissues (massage, stretching, treatment of myofascial trigger points, mobilization, and manual manipulation, combined with physical exercises), which aims to increase the function and to decrease pain and disability in the musculoskeletal system [5]. The treatment concept includes an individually tailored treatment, time to explain the disorders to the patient, and a limited amount of specific home exercises, all of which is believed to have an impact on the long-term effects of the treatment [2,4,18].

### Text Messages as One-Way Reminders

All participants possessed a mobile phone. In the present study, 1 or 2 exercises were given, adapted to the patients’ conditions (ie, stretching of the ilio-psoas and quadratus lumborum muscles, stretching of the glutei muscles, or breathing technique). The most common exercises were stretching of the ilio-psoas muscle and breathing technique. The one-way text messages were individual for each patient, and the exercises were supposed to be performed on a daily basis. The stretching exercises took a couple of minutes each time, while the breathing technique was supposed to be performed now and then throughout the day. The first reminder was sent 3 days after the final visit. As the patients had their first visit at different occasions, spread over almost a year, reminders were sent consecutively, after the patients’ or participants’ last visit. The following reminders were sent every third day for 3 weeks, and then once a week for another 2 weeks (ie, a total of 5 weeks). The treatment sessions normally lasted for 2-3 weeks, which makes an average total time of 7-8 weeks from the first visit to the last reminder. The first message read: “Hello! This is the first reminder of your home exercise(s) (eg, breathing technique and stretching of the ilio-psoas muscle or stretching of the glutei muscles). Please text me to verify that you have received this message. Kindly, xxx.” All the following reminders, except the last, read: “Hello! This is a reminder of your home exercise(s)! Kindly, xxx.” The last message read: “Hello! This is the final reminder of your home exercise(s). I will contact you in 1 week to make a time reservation for the interview. Kindly xxx.”

### Interviews

The participants were asked 2 broad, open-ended questions in a semistructured interview, in order to focus attention on gathering data about the participants’ life worlds (as expressed in phenomenology) and to provide an understanding of the common experiences of the participants [19].

1 *.* “What have you experienced in terms of the phenomenon SMS reminders of home exercises?”

2. “What contexts or situations have typically influenced or affected your experiences of the phenomenon?”

Follow-up questions were guided by the conversations [20]. For example, “What do you mean by that?”; “If I have understood you correctly….”; and “Could you tell a little more about…?”

The interviews were audiorecorded and transcribed verbatim.

### Data Analysis

To understand patients’ experiences of receiving reminders of home exercises after NMT through text messaging, a phenomenological approach with systematic text condensation (STC) according to Malterud was used [20]. A qualitative study was carried out and data were analyzed with STC, deriving from Giorgi’s principles of psychological phenomenological analysis. STC derives from Giorgi’s principles of psychological phenomenological analysis [21]. Phenomenological research can be described as a way to understand the lived relations that human beings have to their world and to other human beings [22]. The reality is comprehended through individual embodied experience and perception, searching for the essence of a phenomenon from the perspective of how it is experienced. It strives to find the participants’ common experience of a phenomenon and significant statements that are valuable. STC is an elaboration of Giorgi’s principles, which includes 4 steps of analysis with specified shifts between decontextualization and recontextualization of data [20]. A limited number of participants [5-15] provide sufficient data for analysis, where the researcher is bracketing his or her presuppositions of the object and moves between identification with, or bracketing, during the different steps of the analysis process.

In the first step, we had an overview of data where the whole transcript was read in order to get a general impression and look for preliminary themes associated with the research question. In the second step, the transcripts were systematically reviewed to identify meaning units. The meaning units were then identified, classified, sorted, and coded according to 3 chosen themes. Data were then reduced to a decontextualized selection of meaning units and sorted into subgroups. The content of the meaning units were thereafter reduced into “a condensate”: an artificial quotation that maintained the terminology used by the participants. A story about the phenomenon with quotations of relevance was then recontextualized. Finally, we searched for data from the transcript that might have challenged our conclusion and compared our findings with existent research findings. We also checked whether our findings challenged our preconceptions.

### Preunderstanding

In this study, the first author’s preunderstanding is based on an empirical perspective; experience of 25 years of clinical work both as an employed and as a privately practicing Naprapath. Initially, the patients consisted of young, elite classical ballet dancers (10-20 years) and later of “ordinary people” both groups adolescents; then, people of working age, older adults, and elderly. The researcher has also educated quality assurance in the Naprapathic core and performed research on treatment and cost effects of NMT at the boundary of specialized care [2,18]. The experience of “doing the right thing from the beginning” (as requested in quality assurance) and of treating patients such as ballet dancers (with very high demands on physical capacity) may have had an impact on the way the information was given to the study participants. Experience of quality assurance may have had an impact in terms of a wish that the participants would perform their exercises thoroughly and continue to perform them even after the study was accomplished. The guiding questions in the interviews might have been influenced by the researcher if, for example, the interviewees would tell that they did not continue to perform their exercises. In order to try to avoid influence from the researcher, there was plenty of time and tranquility during the interviews, in order to give space for the participants to reflect. The researcher also tried to pose few follow-up questions, in order to avoid too much guidance of the participants’ answers.

STC was chosen as it strives for “presenting the experience of the participants as expressed by themselves, rather than exploring any possible underlying meaning of what is said” [20]. This seems to set aside (bracket) the author’s preconceptions as much as possible. The author’s explanatory model was that the participants in this study would find the reminders of home exercises positive, yet a little annoying, disturbing them in their everyday life, and that the reminders would give them a bad conscience about neglected home exercises. The explanatory model was also that the participants would not find the exercises important when the text messages did not arrive anymore or when their pain was gone. All authors discussed the inductive analysis and the emerging themes and any discrepancies between coders. They also refined the analysis and commented on drafts of this article.

### Ethics

The patients in this study were asked for participation at their last visit to the Naprapathic clinic. They received oral information about the study from the naprapath or researcher as well as a written informed consent to be signed if the patient agreed to participate. The patients had the opportunity to have any questions answered and were informed that they had the right to withdraw from the study at any time, without having to state any particular reason and without any negative consequences for them. The audiorecorded interviews and transcripts were kept in locked safes, separated from each other. The research project was approved by the Research Ethics Committee at Lund University (reference no. 2015/494).

## Results

### Results of the Treatment Intervention

The patients in this study had sought this treatment method themselves and it was privately financed, which is not the case for most visits to conventional health care in Sweden. All participants suffered from recurrent LBP and were treated with as many sessions as their condition required (an average of 3 treatment sessions), in order to be free from symptoms. The patients were asked for participation in the study at their last treatment session and were recruited consecutively through purposive sampling that was completed when it was possible to identify themes in the material. The home exercises were thought to help the patients or participants stay pain free and to avoid relapses, and followed normal clinical procedures, in order to aid the transferability of the study.

### Results of the Text Messages as One-Way Reminders

The text messages were sent timely, except for the first message for 2 of the participants. They did not receive this message timely, because they were on vacation abroad, and therefore not connected. Nevertheless, both of them replied a couple of days later, when in Sweden again and when they had received their messages. The text messages were perceived as positive by all the participants. The participants were pain-free when the interviews took place and stated that they did not continue with their exercises because they simply forgot to perform them on a regular basis in between the reminders and after the last reminder was sent. This was also the case if they were on a trip and staying away overnight. During the interviews, though, they reflected about their experiences of the text messages and came to think of, (and planned for), the best way to keep up with the exercises when the test period was over. Three subgroups built up 3 altogether different themes (Table 2).

### Results of the Interviews

Interviews took place a week after the last treatment session (ie, when the SMS reminder would normally have arrived). The interviews were performed by the researcher, in a place chosen by the participants (ie, the Naprapathic clinic) and lasted for 30-40 min each.

**Table 2:** Data analysis.

Themes	Subgroups
Appreciation of the support to perform home exercises	Timely arrival of the text messages
	Usable exercises
	Stimulation to practice memorizing
Reflections about the experiences of the mobile phone text messaging	The aim of the exercises
	The value of the exercises
	Improvement in pain
Creation in order to maintain the improvement	Wishing to adhere to the exercises
	Own routines for the exercises
	The need for extended programs

#### Themes

##### Appreciation of the Support to Perform Home Exercises (Subgroups: Timely Arrival of the Text Messages, Usable Exercises, Stimulation to Practice Memorizing)

The arrival of the text messages was expected by the participants, as they had all been informed about the routines. The messages were perceived as positive and appreciated by all the participants. They found that the exercises for their back were needed and reasoned that what is good for the health is always positive and worthwhile committing to.

To perform the exercises, there is something positive about it. It has only been positive. Everything that affects you positively; at least that’s how I reason; that everything that is positive to yourself, you easily commit to it.

P1

Also, as compared with disturbing advertisements, the messages were not of commercial character, and therefore, rather than being annoying, the reminders were anticipated and appreciated. The participants’ experiences of the reminders were also that they arrived in a timely manner, thus not disturbing.

There is nothing annoying when it comes to such things. It is different with all the telephone salesmen...that is when you get upset! This is only positive. [P5]

The text messages were perceived as easy to handle and the exercises as simple and noninvasive. Therefore, it was possible to perform them as soon as they arrived. As all the participants suffered from recurrent LBP, most of them recognized their given exercises from previous visits in the clinic. It was also appreciated that the exercises were few and perceived as appropriate and effective, and that no equipment was required.

I thought that it was really good to be reminded...it was such an easy exercise, compared with when I was to lay on the floor and pick up a ball and make something that took quite some time; I mean, many more exercises...this exercise, I could perform it when I was standing by the oven, waiting for the tea water to boil.

P3

The participants found that it was helpful to be reminded as they realized that the reminders were necessary if they were to stay pain-free. This made them feel stimulated and motivated them to practice different ways of memorizing to perform their exercises.

Sometimes I had performed my exercise just before the SMS arrived, and then I was very happy that I was ahead, so to say: I had fixed it without the reminder!

P4

I have been eager to perform the exercises before the reminder, since, thus, it is somehow like a reward!

P11

##### Reflections About the Experiences of the Exercises (Subgroups: The Aim for the Exercises, the Value of the Exercises, and Improvement in Pain)

In the last section of the interviews, the participants expressed reflections about the aim of the exercises, how these were to be performed, and in what way they were useful. It seemed that they reflected more on that during the interviews than during the test period.

I haven’t thought of it (performing the exercises), more than, eh, what the aim was; or whether I would feel better, or...I have reflected a little about my breathing, whatsoever, how I breathe (laughter). If I breathe through my belly and how I do that, and when I do that, and when I don’t. Well, I have had these thoughts...you remind me to breathe in a certain way, and then I wonder a little; how do I breathe, actually...I have never reflected on that before

P8

There did not seem to be any doubt that there was a value connected with the exercises. The participants’ experiences were that the reminders did have an impact and that they were valuable.

It does affect you. It does have a value for me. The thing is that it (to perform the exercises) is valuable to me, myself...

P5

Most participants did not suffer from pain or disability when the interviews took place. They stated that, for the time being, they were free—or almost free—from pain, which was somehow surprising to them.

I am a little surprised that it, that my back doesn’t protest, right now. I play extremely much golf, eh, and, sure, I am stiff and so, in the morning, like I use to be, but since I stress my back as much as I do right now, I am a little surprised that it doesn’t protest any more...

P2

The participants were also reflective about the fact that their pain and disability had improved, and they wondered whether there was a connection between the exercises and their improvement in pain.

Well, eh, if I, it is, like I say, I can feel that they actually...that there is something that...I am a little surprised that my back holds...if it is because of those exercises for my belly, I don’t know...

P13

More than forgetfulness, the fact that the participants did not suffer from pain or disability anymore was perceived to be the reason they forgot to continue to perform their exercises. This was also the case when going on a trip and staying away overnight, something that is also often recognized in clinical situations.

Actually, right now my back is fine, and then it is more difficult to remember to perform them (the exercises), as compared with when having pain…when you feel that your back is tired, or when you are in pain, it is a lot easier to remember the exercises...

P7

The thing is that I’ve been away, and then it’s more difficult to remember this. Well, it is quite easy when one is at home, in one’s everyday life...

P10

##### Creation in Order to Maintain the Improvement (Subgroups: Wishing to Adhere to the Exercises, Own Routines for the Exercises, and the Need for Extended Programs)

Some of the participants were curious to know if the intention was that they would continue with the exercises when the study was completed. They reflected about their own commitment to the exercises and how it would be possible to adhere to them in the future in order to maintain their improvement in pain and disability. This became obvious when the intervals between the reminders became longer and finally ceased to come.

In the beginning they (the text messages) came a little more often. Then there were longer intervals, and I ceased to perform my exercises, and when it (the text message) then arrived, I was surprised. By then I had totally forgotten about it. So it...

P8

I don’t claim that I have a routine. I perform the exercises whenever I come to think of them, and it...(laughter) one should have it as a routine, actually; a couple of times every day.

P6

Most participants presumed that the exercises should be carried out continuously even when the pain was gone, and they reflected on how it would be possible to make them become a natural and smooth part of their everyday life. When they reflected on the need for continuation, they came to think of quite different solutions to create their own routines. The solutions varied depending on what their everyday lives and routines looked like—if they visited a gym, or a golf club, for example, or if they mostly spent their days at home. They came to think of having specific routines when going to the gym or when warming up before a golf session to perform the exercises at the same time as their daily medication, set mobile phone alerts, or write a diary for the exercises. All in order to make it possible to continue to perform the exercises when the test period was over.

But well, I do have certain routines...so it would be...if I would make it a routine, for example in the beginning of each golf session, when I am warming up. There I think that I would do it. Because I use to, eh, try to stretch my back before starting to swing. And there I would think that I could perform those exercises too, at the same time. I would consider that! But not otherwise; you have to connect it to something.

P2

...one should have it as a routine, actually; a couple of times each day. One should actually have them at each time. “Well, now I have to do it.” That it says “pling” and then I have to do them. Of course, this would be possible for me to arrange myself; I have an alert on, in order to take a pill, at a certain time and...I have it continuously, that alert, every day. So I would be able to fix that on my own.

P5

...I thought then that one alternative to this would be to make a list and to tick it off, and...that you make your own list; that wouldn’t be bad, because thus I’d see: “well, I didn’t do anything yesterday.

P3

Some of the participants even figured that additional exercises would make it possible that they would avoid relapses and to stay pain-free overall, and this is why they requested extended exercises. Thus, it seemed that their creativity was stimulated.

There are many exercises that strengthen the back, for example. It would have been nice with some more...one does need some extra practice. More in general, exercising the back and so on...one would need to take action on thatBecause one shouldn’t have to be in such pain, because of making a movement that the body is not used to. If you are sufficiently well trained, it shouldn’t hurt.

P1

I associated the exercises in the text messages with other exercises that I have performed previously, so I did all of them when the reminders arrived.

P14

## Discussion

### Principal Findings

The main finding of this study was that reminders in the form of mobile phone text messages for home exercises for elderly patients who have received manual therapy for recurrent LBP were appreciated. It was perceived as positive and valuable to be reminded of home exercises and to practice memorizing. The participants reflected about the aim and the value of the exercises, and in order to maintain their improvement they created their own routines for continued adherence *.*

### Discussion of the Results

There is only a limited amount of published studies that have evaluated text messaging as a method to promote physical activity [23]. Few studies (9 RCTs with small sample sizes) on patients with chronic LBP show reduced catastrophization and improved patient attitudes when using Web-based interventions like, for example, Web-based cognitive behavior therapy (CBT) [24]. These studies contain interactive components and they are quantitative, focused on outcomes, not qualitative (ie, focused on the participants’ experiences), and that makes it difficult to compare their outcomes with the outcomes of this study.

The strengths of this study are that the results were distinct, and that the text messages were perceived as positive, simple, and timely. The participants in the present study were pain-free when the interviews were performed, and this is in line with earlier research with positive outcomes in studies where text messaging was used [11-13]. The participants in our study had chosen a private, complement (specialized manual therapy in the shape of Naprapathic manual therapy), and therefore, they may have been extra motivated to limit the number of treatment sessions, as they were expensive, compared with treatments in the reimbursed national health care system. Yet, the intention of our study was to explore how the text messages were perceived, not to measure the effectiveness of the treatment.

A major factor that contributes to increase the adherence to expert advice, according to the concept of health literacy, is improving people’s understanding of what is provided in the realm of medical services [25]. Accordingly, the participants in our study were knowledgeable of the treatment option NMT (not available in the national health care system) and had made an active choice, which is a strength. They were thereby also “selected,” and maybe more motivated to adhere to the exercises, which is a weakness with regard to the transferability to other groups of patients.

The exercises were perceived as simple, and during the interview the participants reflected on the aim and the value of the exercises and on the fact that the pain was gone. We believe it was because of the exercises because of earlier studies on the effects of text messages, which have concluded that the outcomes of such interventions, in terms of medication adherence, clinical management, and health-related behavior modification are positive changes and significant improvements [13,22].

Meanwhile, the participants realized that they easily forgot to perform the exercises when the reminders ceased to arrive or when the pain was gone. As they found that the exercises were valuable and they appreciated the memorizing practice, they reflected and created their own routines for remembering the exercises to avoid relapses of pain. This is probably the most salient and valuable finding of this study, and it is supported by earlier research, where patient participation and behavior change were important parts of improved self-management in chronic health disorders [26]. This is also in line with another health concept, namely, patient empowerment. The starting point of patient empowerment is that people may improve their health by controlling the conditions that rule health [27]. Positive outcomes of Web-based interventions for patients with chronic LBP in terms of increased patient empowerment and coping strategies have been found in several earlier studies [24]. The choice of treatment and research techniques utilized in this study are strengths, as they have not been studied before. Also, they embrace both health literacy (making an active choice) and patient empowerment (home exercises and reflection about adherence), which may improve peoples’ health.

This internalization may be difficult to transfer to if only using text messages (no interviews) though, as it may be that the participants’ reflection and creation emerged as a result of the interview. Somebody was interested in the participants’ opinions and thoughts; they had a lot of time to reflect during the interview and were being listened to. Previous studies have concluded that text messages combined with other delivery approaches, that is, “face-to-face” interviews and implementation intentions planning in advance are significantly more effective for changing health behavior than one method only [28-30]. Therefore, an important question is, “What will make the participants continue with their exercises when they do not suffer from pain or dysfunction and do not receive the reminders anymore”? Will “creation in order to maintain the improvement” and adherence to the exercises be possible without the interviews, where the participants’ creativity emerged [28]?

Patient participation and behavior change may also be easier to achieve when turning to older adults, as their health is more vulnerable compared with younger adults, and because they have a less-stressful everyday life than the working population. They probably have the time to reflect on the exercises and are also probably more motivated than younger people to practice something that stimulates the memory. However, a previously published study on the effects of reminders through text messaging concluded that text messaging was a tool for behavior change across all ages [11]. More rigorous, theory-based intervention research in pediatric and adolescent populations is needed, though [12].

### Discussion of the Method

A phenomenological approach and an inductive method were chosen, in order to try to capture the essence of the participants’ own experiences as much as possible and what they have in common, and to avoid interpretation of any underlying, latent meanings from the researcher. Looking for similarities might have biased the study though, as the interviewees were all very positive to the phenomenon. Yet, that was not known until the interviews were carried out. Strengths with the study are that the research question is new, the sample was chosen from the “real world” and of almost equal numbers from both genders. Also, it comprised elderly, which is an increasing group of patients, yet not often included in trials.

As mentioned earlier, the sample of participants in the present study was selected. Apart from being older, they had also sought a private clinic for their LBP, which may make it difficult to evaluate the transferability of this study to other contexts, such as hospital settings, for example, to which patients are referred.

The standards with regard to the frequency and duration of the text messages vary compared with former studies (from several times a day to once a month, and for 3-12 months, respectively [11,13]). In this study, the intervals between the reminders were chosen pragmatically; initially they were the same as the intervals between the first and the second treatment sessions (ie, 3 days). This may be considered a weakness; the internalization might have been more evident if the reminders would have arrived more frequently and the test period had lasted longer (ie, more than 5 weeks), yet this has not been confirmed in any previous study. The manual therapist and the interviewer were one and the same person in this study, and the participants are also patients. These are also weaknesses, as patients may want to please their therapist, which increases the risk of bias. Also, the (active) role of the manual therapist or researcher may have an impact when it comes to the patients’ reflection and creation. Yet, the method (STC) used in this study appreciates that the researcher in the final analysis reflects on whether the findings challenge the researcher’s preconceptions [20]. In this study they did; the participants were expected to find the reminders a little annoying, and their reflection and creation were not expected, which contribute to the reflexivity of the study, hence a strength.

The reminders made the patients reflect on their exercises and why they were pain-free and increased their understanding of sustainability in health. Thus, the study has clinical relevance. It also has technical implications, in that the method is widely available, cheap, and easy to start up, which has also been found before [8-11], and it is possible to elaborate the messages with extended and individually tailored exercises, for example. There is also the possibility of using text messaging the other way around, as found in previous studies, in order to enhance long-term follow-ups in clinical trials [31]. This study may serve as a small, yet an important contribution when striving to find methods that may have an impact on the long-term effects of an intervention.

Further research is needed, both with a technical focus (how often and for how long is it necessary for the text messages to arrive in order for patients to internalize their exercises, and if similar results are possible without interviewing patients?) and with a clinical focus (is it plausible that SMS reminders of home exercises may translate into improved long-term effects of manual therapy?)

### Conclusions

Mobile phone text messages as reminders of home exercises after manual therapy for recurrent LBP in the elderly were appreciated among the interviewed study participants. The reminders made the participants reflect about the aim and the value of their exercises and of their improvement in pain. They appreciated that they had to practice memorizing and, in order to maintain their improvement during the interviews they created their own routines for adherence to the exercises. Thus, it seems probable that mobile phone text messaging may serve as a useful tool for patient empowerment with regard to recurrent LBP in older persons.

## Abbreviations

CBT: cognitive behavior therapy
LBP: low back pain
NMT: naprapathic manual therapy
SMS: short messaging service
STC: systematic text condensation
